# Advancing science and adhering to ethics—Mission and practice of CALAS

**DOI:** 10.1002/ame2.70137

**Published:** 2026-01-12

**Authors:** Chinese Association for Laboratory Animal Sciences

## Editorial Message: Asia Laboratory Animal Day: Origin and Founding Mission—Paying Tribute to the Silent Contributors in Scientific Research

In the journey of advancing human scientific progress and health well‐being, laboratory animals serve as indispensable silent contributors. To commemorate their contributions and advocate for welfare principles, the “Asia Laboratory Animal Day” was established. This initiative is the result of collaborative efforts among various laboratory animal organizations across Asia, forming a crucial link in connecting scientific research ethics and humanistic care throughout the continent.

The establishment of “Asia Laboratory Animal Day” is a testament to the deep collaboration and shared vision of member organizations within the Asian Federation of Laboratory Animal Science Associations (AFLAS). During the 2023 Annual Academic Conference on Laboratory Animal Science and Technology of China, the proposal to “establish Asia Laboratory Animal Day” received enthusiastic responses from AFLAS member organizations. These organizations engaged in in‐depth discussions on laboratory animal welfare, fostering broad consensus. In September of the same year, at the AFLAS Council Meeting held in Seoul, South Korea, representatives from member organizations delved into the topic, drawing on practical experiences to offer suggestions and propel the initiative forward with strong agreement. In September 2024, following deliberation by the AFLAS Council, the founding day of AFLAS—November 29th—was officially designated as “Asia Laboratory Animal Day.”

The core intentions behind establishing “Asia Laboratory Animal Day” encompass multiple dimensions. Firstly, it is to express sincere gratitude to laboratory animals that silently contribute to life science research. With their irreplaceable value, laboratory animals provide critical support for fields such as medicine and pharmacy within the life sciences, playing an indispensable contribution in driving theoretical innovations and technological breakthroughs. Secondly, the “Asia Laboratory Animal Day” serves as an opportunity to deepen the promotion and practice of laboratory animal welfare principles. It aims to advance the implementation of the 3Rs (Replacement, Reduction, Refinement) principles alongside the newly added 4th R, “Responsibility,” while simultaneously improving the regulatory framework for laboratory animal management. Relying on technological innovation, it seeks gradual breakthroughs in scientific substitutions. Thirdly, through annual themed commemorative events, it establishes a bidirectional enhancement mechanism for social awareness and scientific ethics. This enhances societal recognition of the importance of laboratory animal welfare, promotes scientific moral education, and commemorates the contributions of laboratory animals to scientific progress. Lastly, it provides a platform for exchange among Asian countries and regions regarding laboratory animal welfare, enabling the sharing of best practices and jointly upholding research quality and ethical standards.

Now, as “Asia Laboratory Animal Day” enters its second edition, its commemorative significance, welfare principles, and spirit of cooperation are transcending national borders, gathering more attention and actions to ensure that scientific progress and life care proceed hand in hand.

## Advancing science and adhering to ethics—Mission and practice of CALAS

### Chinese Association for Laboratory Animal Sciences

On the occasion of “Asia Laboratory Animal Day,” we would like to express our highest respect to laboratory animals, the “silent contributors,” for their tremendous contributions to human health and scientific progress. The Chinese Association for Laboratory Animal Sciences (CALAS) has always been committed to promoting the development of laboratory animal science, enhancing animal ethical welfare, popularizing scientific knowledge, and shouldering the responsibility of continuously strengthening public education and actively advancing the progress of laboratory animal science and technology.

As one of the founding and member associations of the Asian Federation of Laboratory Animal Science Associations (AFLAS), CALAS has long been devoted to the development of laboratory animal science and technology and the building of multidimensional academic exchange platforms. Focusing on themes like “Laboratory Animal Science and Technology and Human Health,” we have successfully hosted widely influential international summits, such as the 3rd and 10th AFLAS Congresses. These events have established a stage for academic dialogues across countries and disciplines, injecting new momentum into the cooperation and sharing in the Asian region and the world at large.

CALAS attaches great importance to the welfare and ethics of laboratory animals and has established a series of top‐level designs and management standards, achieving both standardized and systematic ethical framework. First, the standard system has been increasingly refined. CALAS has issued a total of 167 group standards and created a comprehensive system of standards in laboratory animals, and in the management, technical procedures, and personnel training of animal experiments. Second, ethical supervision has been thoroughly strengthened. Initially proposed by CALAS, the “Competence Evaluation System of Laboratory Animal Institutions” has led the industry to a more standardized direction. With “Regulate” at its core, CALAS has formed a closed‐loop, sustainably perfected management system that prioritizes standards, leads with ethics, and ensures regulatory oversight in order to implement the “3Rs” principle centered on Replacement, Reduction, and Refinement.

It is essential to upgrade the technician‘s level of technique and ethics if we want to implement and fulfill the welfare and ethics requirements. Therefore, CALAS has launched the “Laboratory Animal Veterinarians Training and Certification” and constituted a standardized, nondegree vocational education and certification system in the field of laboratory animal medicine in China. Furthermore, it has introduced the “Laboratory Animal Technician Competency Evaluation” system, forming a whole certification chain, which completely improves the professional skills and ethical awareness of practitioners.

**FIGURE 1:**
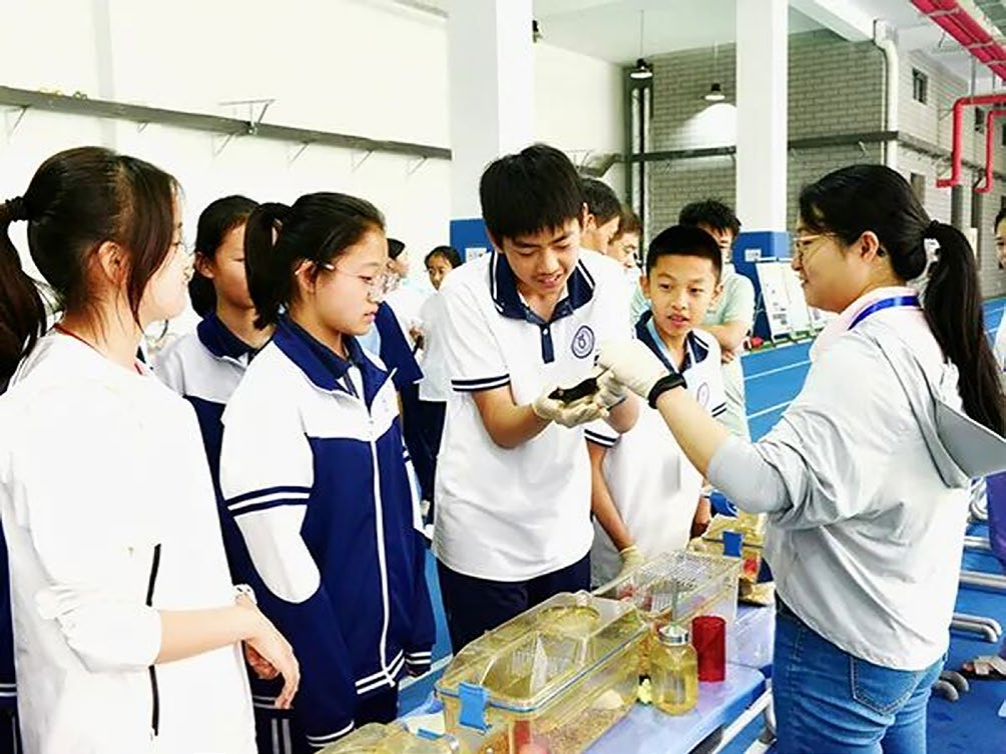
“The Light of Life in the Laboratory” — National Science and Technology Week 2025: Science Popularization Outreach Activities on Campus.

CALAS has also established a science popularization system toward the general public through science education campaigns. It not only organizes experts to engage with schools and communities, but also promotes the deep integration of life sciences and ethics education through lectures and interactive experiences, enhancing public awareness—especially among the younger generation—of laboratory animal science and technology (Figure 1). 

**FIGURE 2:**
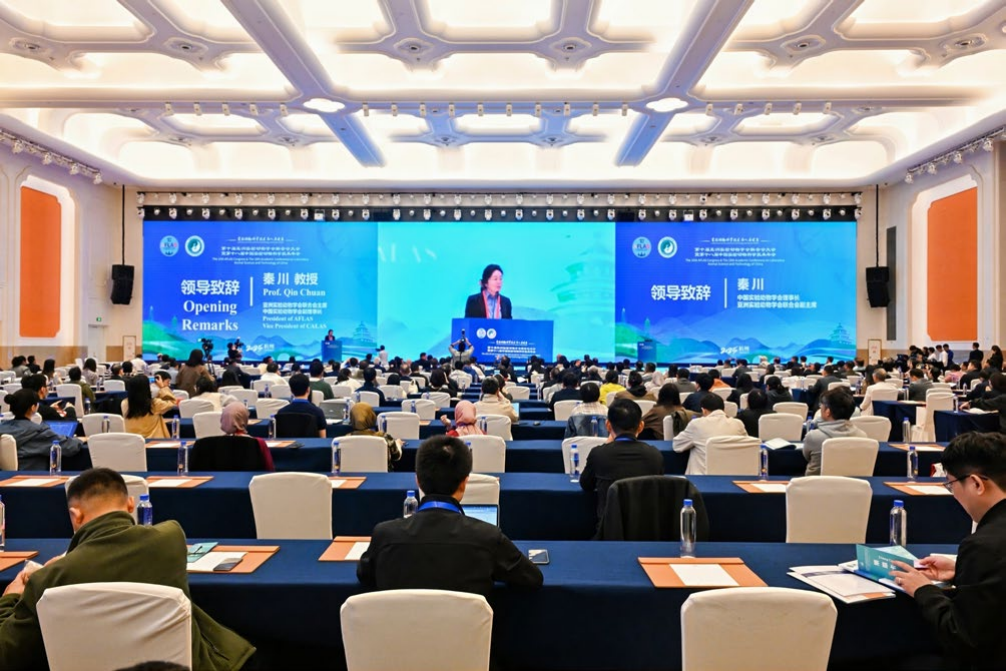
Professor Qin Chuan, President of the Asian Federation of Laboratory Animal Science Associations (AFLAS) and Vice President of the Chinese Association for Laboratory Animal Sciences (CALAS), delivers a speech at the opening ceremony of the 10th AFLAS Congress.

Professor Qin Chuan, President of AFLAS and Vice Chairperson of CALAS, once proposed that standardized management is an important approach to ensuring animal welfare and ethics (Figure 2). It requires us to learn international experiences in accordance with Chinese cultures, and seek development grounded in our own cultural characteristics. We have always upheld an open and inclusive cultural foundation, and strived to improve the resource development and sharing of animal models, reach an internationally recognized consensus in terms of laboratory animal technical and welfare standards featuring mutual learning and inclusiveness so as to foster a shared development.

## AUTHOR CONTRIBUTIONS

**Chinese Association for Laboratory Animal Sciences:** Writing – original draft; writing – review and editing.

